# Multi-Omics Analysis of the Potential Mechanisms of Skin Albinism in Edangered *Percocypris pingi*: Abnormal Ubiquitination and Calcium Signal Inhibition

**DOI:** 10.3390/cells14211684

**Published:** 2025-10-27

**Authors:** Senyue Liu, Xiaoyun Wu, Qiaolin Zou, Jiansheng Lai, Luyun Ni, Yongqiang Deng, Yang Feng, Mingjiang Song, Pengcheng Li, Jun Du, Qiang Li, Ya Liu

**Affiliations:** 1 Fisheries Research Institute, Sichuan Academy of Agricultural Sciences (Sichuan Fisheries Research Institute), Chengdu 611731, China; liusenyue@scsaas.cn (S.L.);; 2 Aquatic Health and Intelligent Aquaculture Key Laboratory of Sichuan Province, Chengdu 611130, China

**Keywords:** albinism, ubiquitination, calcium signaling, multi-omics, pigment

## Abstract

*Percocypris pingi* is an endangered protected fish species in China. Its albino variants exhibit growth retardation and physiological abnormalities. Understanding its albinism mechanism holds significant scientific importance for molecular breeding programs and disease model development. This study integrated transcriptomic and proteomic analyses, combined with histopathological and molecular biological techniques, to systematically compare molecular differences in skin tissues between albino and wild-type *P. pingi*, with a focus on elucidating the multidimensional regulatory mechanisms underlying skin albinism. Our findings suggest that albinism in *P. pingi* is synergistically driven by hyperactivation of ubiquitin-mediated proteolysis (which suppressed TYR/TYRP1 enzymatic activity and disrupted the pH homeostasis of melanosomes), and inhibition of calcium signaling (which impeded melanin transport). This discovery provides novel insights into the mechanisms of pigment loss in fish species and offers a valuable reference for molecular breeding of endangered species as well as research on pigmentation-related disorders.

## 1. Introduction

In vertebrates, melanin biosynthesis initiates within specialized membrane-bound organelles called melanosomes within melanocytes [1]. Serving as the primary sites for pigment deposition, melanosomes undergo directional translocation via the cytoskeletal system to neighboring keratinocytes following melanin synthesis, thereby driving skin pigmentation diversity [2]. Melanin production, as the central biological process governing pigment synthesis, proceeds via precisely coupled enzymatic and chemical reactions [3,4], exhibiting critical dependence on the homeostasis of tyrosinase (TYR) family enzyme activity, efficient melanosome trafficking, and balanced intracellular ion microenvironments [5]. The melanogenic cascade begins with the TYR-catalyzed oxidation of tyrosine to dopaquinone. Through the coordinated actions of key enzymes including TYRP1, this pathway bifurcates to produce two distinct melanin classes: eumelanin (EM, black-brown) and pheomelanin (PM, yellow-red). The relative abundance and spatial distribution patterns of EM and PM collectively constitute the mechanistic basis for vertebrate pigmentation diversity [3].

Albinism, a canonical pathological phenotype resulting from disruption of pigment cell developmental programs in vertebrates, has long been the research focus of developmental biology and pathology. This phenotype stems from defective melanogenesis pathways causing reduced melanin production [6], involving multi-dimensional networks such as melanin biosynthesis, proteostasis, ion transport, and endocrine signaling [7]. In mammals, ubiquitin–proteasome system (UPS)-mediated degradation of tyrosinase family proteins [8,9,10] and calcium signaling-regulated melanosome trafficking networks [1,11,12,13] have been confirmed as the core regulatory nodes governing albinism. However, as a pivotal evolutionary branch of vertebrates, there are still significant knowledge gaps regarding albinism mechanisms in teleost fishes, and the specific regulatory network remains elusive.

*Percocypris pingi* was listed in China’s Vertebrate Red List in 2015 [14] and is recognized as a critically endangered fish species under national protection, making the conservation of its wild populations and genetic improvement scientifically imperative [15]. During artificial breeding of *P. pingi*, the Chinese aquaculture industry has identified two distinct color species: a wild-type (WT) exhibiting gray-black coloration with speckled patterning, and rare albino individuals demonstrating complete loss of skin pigmentation. These albino individuals consistently exhibit slower growth than their WT counterparts with concurrent physiological abnormalities. Consequently, elucidating the regulatory mechanisms underlying albino skin development in *P. pingi* is of great significance for assisting in the molecular marker breeding of endangered carp species and the development of related disease models.

Consequently, this study employed *P. pingi* as the model organism to systematically analyze the underlying mechanisms of skin albinism by integrating transcriptomic and proteomic analysis and combining histopathology and molecular biology, thereby establishing mechanistic foundations for understanding pigment loss and associated physiological anomalies in teleost fishes.

## 2. Materials and Methods

### 2.1. Experimental Fish

All experimental *P. pingi* were obtained from a commercial artificial breeding population at an aquaculture facility in Mabian County, Leshan City, Sichuan Province, China. Sixteen healthy albino *P. pingi* individuals (designated group W, where “W” stands for “White” to denote the albino phenotype) and eight wild-type *P. pingi* individuals (designated group C, where “C” stands for “Control” to denote the wild-type phenotype) were utilized (each group consists of eight biological replicates).

Both albino and wild-type fish originated from the same population and had been co-cultivated long-term under identical environmental conditions (water temperature at 22 ± 1 °C, pH = 7.2 ± 0.3, and dissolved oxygen > 6 mg·L^−1^) and standardized photoperiod conditions (light:darkness = 12 h:12 h) to eliminate environmental confounding factors. The albino individuals were naturally occurring mutants identified during routine breeding. The key biological characteristics of the fish were as follows: all individuals were 11-month-old juveniles, at which stage the albino phenotype (complete skin depigmentation) and wild-type phenotype (gray-black skin with speckles) were stably distinguishable. The biological indicators of the wild-type fish (group C) are an average weight of 8.73 ± 1.24 g and an average body length of 8.36 ± 0.45 cm, while those of the albino fish (group W) are an average weight of 6.21 ± 1.62 g and an average body length of 7.43 ± 0.76 cm.

### 2.2. Sample Collection

Following immersion anesthesia using buffered MS222 (250 mg·L^−1^; Aladdin, Shanghai, China), the biological parameters of the two groups were measured (*n* = 8). Twelve randomly selected samples (six fish in each group) and skin tissues were collected from the lateral line scale area (0.5 cm^2^) under aseptic conditions. Some of the collected tissue samples underwent immediate fixation in a 4% paraformaldehyde solution for subsequent histopathological evaluation (*n* = 3), while the other part was cryopreserved at −80 °C for subsequent molecular analysis (*n* = 3).

### 2.3. Histological Analysis

#### 2.3.1. Hematoxylin and Eosin (H&E) Staining

Skin tissues from both groups underwent sequential processing: paraffin embedding, sectioning, H&E staining according to established protocols [16], and final sealing with neutral resin. Pathological alterations within sections were subsequently assessed and documented utilizing a Nikon Eclipse E200 microscope (Tokyo, Japan) (*n* = 3).

#### 2.3.2. Fontana–Masson Staining

Melanin staining was carried out according to the established method [17]. Following deparaffinization and rehydration, tissue sections were incubated with Fontana–Masson silver stain at 37 °C for 45 min. Subsequent treatment included formalin reduction and gold chloride differentiation, followed by nuclear counterstaining with hematoxylin. Sections were cleared in xylene, sealed with resin, and examined under a Nikon Eclipse E200 microscope (Black: melanin/argentaffin cells; Red: collagen; Yellow: muscle fibers/RBCs). For melanin quantification, five non-overlapping fields (200× magnification) were randomly selected from each section; images were analyzed via ImageJ (v2.0) to calculate the percentage of melanin area (black-stained regions) relative to total skin area. (*n* = 3).

#### 2.3.3. Immunohistochemistry

The paraffin sections were deparaffinized to water and subjected to antigen retrieval using a boiling sodium citrate solution (pH 6.0, 10 mM, Servicebio, Wuhan, China). Non-specific binding and endogenous peroxidase were blocked with 3% hydrogen peroxide (H_2_O_2_), followed by 30 min room-temperature blocking with 3% bovine serum albumin (BSA, Thermo Fisher Scientific, Waltham, MA, USA). Sections were incubated overnight at 4 °C with anti-UBE2J1 Rabbit pAb (1:400, GB111868, Servicebio) and anti-CACNG1 Rabbit pAb (1:500, GB111624, Servicebio) in a humidified chamber, then incubated with 1% HRP-labeled goat anti-rabbit IgG (Servicebio) for 50 min at room temperature. After DAB staining, nuclei were counterstained, dehydrated, and mounted. Then, observation and photography were conducted under a Nikon Eclipse E200 microscope for analysis. The positive results appear brownish-yellow. (*n* = 3).

### 2.4. RNA Isolation and Qualification

Adhering to the manufacturer’s protocol, total RNA was isolated from skin tissues (*n* = 3) employing Trizol reagent (Takara Bio, Ohtsu, Japan). The extracted total RNA underwent a comprehensive quality assessment: the ratios of A260/A280 and A260/A230 were determined by spectrophotometry (NanoDrop2000, Thermo Fisher Scientific, Waltham, MA, USA); structural integrity was evaluated by agarose gel electrophoresis; and the RNA integrity number (RIN) was determined using the Agilent 2100 bioanalyzer (Agilent Technologies, Santa Clara, CA, USA). Only samples meeting the criteria (Total RNA ≥ 1 μg, concentration ≥ 45 ng μL^−1^, A260/A280: 1.8–2.2, A260/A230: 2.0–2.2) proceeded to library construction.

### 2.5. Transcriptomic Profiling

#### 2.5.1. Library Construction and Sequencing

Illumina-compatible sequencing libraries were synthesized from qualified mRNA. Paired-end sequencing was executed on an Illumina NovaSeq X Plus platform (Gene Denovo Biotechnology Co., Ltd., Guangzhou, China). Raw sequence data underwent initial quality evaluation using FastQC (Cambridge, UK) to ensure reliability for downstream analyses.

#### 2.5.2. Raw Data QC, De Novo Assembly and Functional Annotation

Raw reads were filtered with fastp (v0.18.0) for quality control. Specific criteria included: (1) removing reads containing adapter sequences; (2) filtering out reads with >10% unknown bases (N); (3) excluding reads with excessive adenine (A) content (entirely A-based reads); (4) trimming low-quality reads (where bases with quality score ≤20 accounted for >50% of the total length).

*De novo* assembly of high-quality reads was conducted using Trinity software (v2.8.4) [18]. The resulting unigene sequences were subjected to homology-based functional annotation via blastx alignment (E-value threshold < 1 × 10^−5^) against the Gene Ontology (GO, http://www.geneontology.org/) (accessed on 15 April 2025) and Kyoto Encyclopedia of Genes and Genomes (KEGG, https://www.genome.jp/kegg/) (accessed on 15 April 2025) databases.

#### 2.5.3. Gene Expression Quantification and Normalization

Unigene expression abundance was quantified via RSEM software (v1.3.2) [19], generating raw read counts, and FPKM. The FPKM matrix containing normalized expression values of all genes across all samples (three replicates per group) is provided in Supplementary Table S1. Inter-sample relationships were explored through principal component analysis (PCA) executed in R (version 4.1.0).

#### 2.5.4. Differential Expression and Enrichment Analysis

Differential expression analysis was performed using edgeR (v3.9.0), which employs a negative binomial distribution model. Significantly differentially expressed genes (DEGs) were defined by stringent thresholds: |log_2_FC| > 1.5 and false discovery rate (FDR) < 0.05, with FDR correction via the Benjamini–Hochberg method [20]. The complete list of DEGs is provided in Supplementary Table S2. Functional enrichment analysis of DEGs was conducted against the GO Consortium database (release 20160301) and KEGG Animal pathway database (release 93.0) using the hypergeometric test. Significantly enriched terms were defined as those with *q*-value < 0.05 and enrichment factor > 1.5.

### 2.6. Proteomic Profiling

#### 2.6.1. Sample Processing and Mass Spectrometry Acquisition

Protein profiling was conducted using an Orbitrap Exploris 480 platform (Gene Denovo Biotechnology Co., Ltd., Guangzhou, China). Tissue lysates underwent preparation with the iST sample preparation kit (PreOmics, Munich, Germany), encompassing protein extraction, denaturation, disulfide bond reduction and alkylation, tryptic digestion, and peptide desalting. FDR thresholds were set to 1% for precursor ions, peptides, and proteins to ensure identification accuracy. Liquid chromatography-tandem mass spectrometry (LC-MS/MS) analyses were executed on an EASY-nLC 1200 system coupled to an Orbitrap Lumos mass spectrometer (Thermo Fisher Scientific). Spectral data were acquired in data-independent acquisition (DIA) mode, collecting only mass spectra (m/z and signal intensities).

#### 2.6.2. Protein Identification, Quantification, and Functional Classification

Raw spectral data were processed using Spectronaut X software (v19.7, Biognosys AG, Zurich, Switzerland) against the Uniprot database. Search parameters included fixed modification (carbamidomethylation of cysteine) and variable modification (oxidation of methionine). Protein quantification was based on normalized abundance using Intensity-based absolute quantification (iBAQ), with reproducibility assessed via coefficient of variation (<20% across replicates). Functional annotation utilized GO, KEGG, KOG, Swiss-Prot, NR, and Animal TFdb databases.

#### 2.6.3. Sample Relationship Analysis and Differentially Expressed Proteins (DEPs)

The inter-sample relationship was evaluated through PCA implemented within the R statistical environment. DEPs were identified using a two-step statistical model: raw protein intensities were compared via Student’s *t*-test, followed by FDR correction via the Benjamini–Hochberg method. Significance thresholds were set at: |log_2_FC| > 1.5 and FDR < 0.05.

#### 2.6.4. Functional Enrichment Assessment

DEPs were mapped against the GO and KEGG database (GO Consortium and KEGG Animal pathway). Enrichment significance was computed using the hypergeometric test, with terms considered significant at *q*-value < 0.05 and enrichment factor > 1.5.

### 2.7. Gene Set Enrichment Analysis (GSEA)

To identify significantly enriched biological pathways, GSEA was conducted employing GSEA software (v4.2.3) with GO and KEGG databases. Specifically, RNA-seq data were normalized using FPKM, and proteomic data using iBAQ. Genes/proteins were ranked by Signal2Noise metric ((μa − μb)/(σa + σb)),where μ is mean expression and σ is standard deviation. Permutation testing (1000 permutations) was used to compute raw *p*-values, with FDR correction via Benjamini-Hochberg. Advanced statistical methods were employed: edgeR for RNA-seq DEGs, limma-voom for proteomic DEPs, and permutation-based GSEA.

### 2.8. Validation via Quantitative Reverse Transcription PCR (qRT-PCR)

Technical validation of transcriptomic findings and assessment of target gene expression profiles were performed through qRT-PCR (*n* = 3). Primers were designed using Premier 6 software (sequences detailed in Supplementary Table S3). Amplification reactions employed protocols adapted from established methodologies [21], with thermal cycling parameters and reaction volumes configured accordingly. The assays were performed on a CFX96 Touch™ Real-Time PCR System (Bio-Rad, Hercules, California, USA). Threshold cycle (Ct) values for *P. pingi* transcripts were normalized against the internal reference gene e*EF1-α* and *β-actin* [21,22]. Relative quantification of target gene expression was calculated according to the 2^−ΔΔCT^ method [23].

### 2.9. Statistical Analysis

All experimental data were presented as mean ± standard deviation. Prior to inferential analysis, normality and homogeneity of variance were assessed using the Shapiro–Wilk test and Levene’s test, respectively. The significance threshold was set at *p* < 0.05. Specific analytical methods for each data type were applied as follows: omics-derived differential genes/proteins (DEGs/DEPs) were identified according to the criteria in Section 2.5.4 (transcriptomics, edgeR negative binomial model) and Section 2.6.3 (proteomics, two-step *t*-test), with FDR correction via the Benjamini–Hochberg method; relative gene expression from qRT-PCR was calculated using the 2^−ΔΔCT^ method and compared by Student’s *t*-test; quantification of melanin was analyzed with ImageJ (v2.5.0) and evaluated by Student’s *t*-test; GSEA was performed using normalized expression data (FPKM for transcriptome, iBAQ for proteome), applying the Signal2Noise metric and 1000 permutations with an FDR threshold of <0.05. Figures were generated with GraphPad Prism 9.0 (San Diego, CA, USA) and Adobe Illustrator CC 2023 (San Jose, CA, USA).

## 3. Results

### 3.1. Clinical Phenotype and H&E Staining

**Figure 1:**
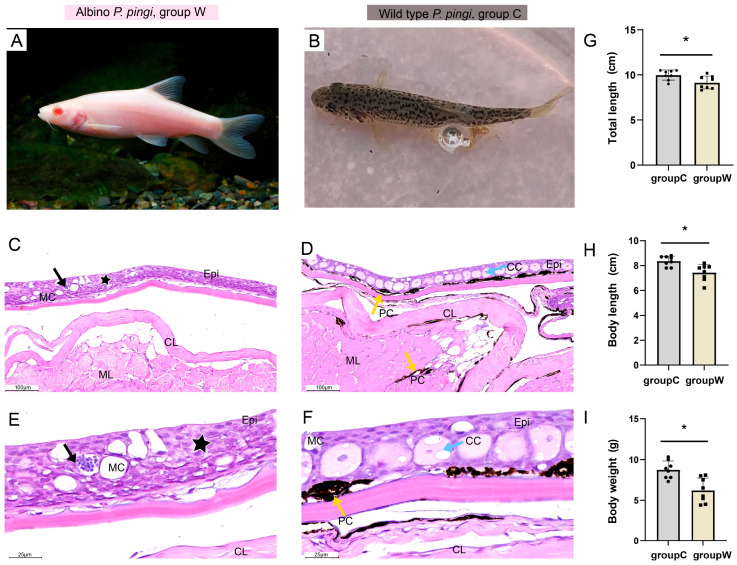
Clinical phenotypes and H&E staining. (**A**) Clinical presentation of group W (1×). (**B**) Clinical presentation of group C (1×). (**C**–**F**) H&E staining results (*n* = 3): (**C**) H&E-stained skin section from group W showing complete absence of melanin granules and epidermal necrosis (star). (**D**) H&E-stained skin section from group C demonstrating intact epidermis and abundant melanin granules (yellow arrows). (**E**) Albino skin section exhibiting epidermal necrosis (star) with concomitant inflammatory cell infiltration (black arrows). (**F**) Control skin section containing dense melanin aggregates (yellow arrows). (**G**–**I**) Morphometric parameters of *P. pingi*: (**G**) total length, (**H**) body length, and (**I**) body weight (* *p* < 0.05; *n* = 8). MC, mucous cells; CC, club cell; Pc, pigment cell; Epi, epidermal layer; CL, compact layer; ML, muscle layer.

Clinically, albino *P. pingi* exhibited a characteristic generalized depigmentation phenotype, presenting uniform white skin devoid of speckling (Figure 1A), whereas wild-type displayed dark body color with regular spotting patterns (Figure 1B). Growth performance analysis revealed significantly lower total length, body length, and body weight in the albino *P. pingi* compared to wild-type individuals (Figure 1G–I). This growth retardation is likely attributable to energy metabolism dysregulation associated with pigment synthesis disorders.

Histopathological examination via H&E staining revealed the complete absence of melanin granules throughout the epidermal layer, dermis, and subcutaneous tissues in albino *P. pingi* (Figure 1C), with occasional lymphocyte infiltration and epithelial cell necrosis observed in the epidermis (Figure 1E). In contrast, wild-type exhibited an intact skin epithelial layer with normal cellular morphology (Figure 1D), displaying abundant melanin granules distributed both within the epidermis–dermis, as well as the dermis–subcutaneous junction (Figure 1F).

### 3.2. Transcriptome Differential Analysis and Proteome Differential Analysis

**Figure 2:**
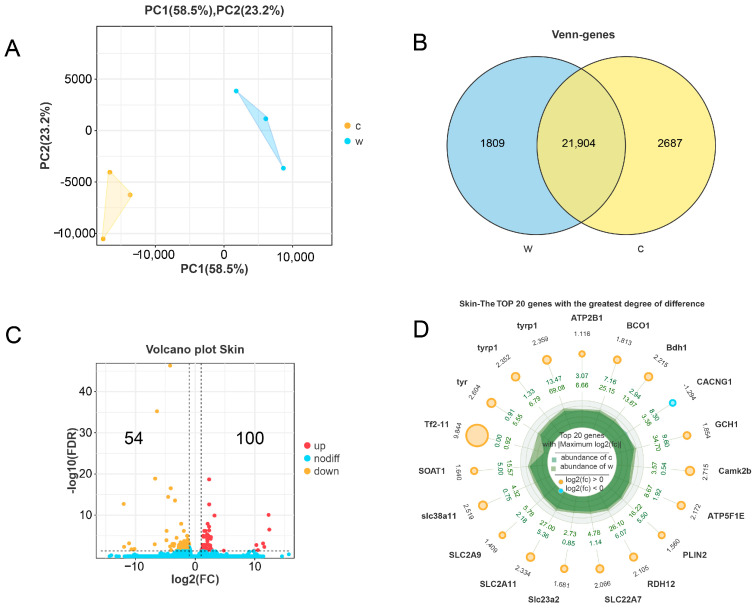
Relationship analysis and basic differences analysis. (**A**) Principal component analysis (PCA) of two groups. (**B**) The Venn diagram shows common genes and specific genes between two groups. (**C**) The volcano plot shows the distribution of DEGs between two groups. (Red dots indicate up-regulated DEGs, orange dots indicate down-regulated DEGs, and blue dots indicate genes with no difference). (**D**) Visualization of the top 20 genes with the greatest degree of difference ranked by *p*-value between two groups. The concentric rings represent: inner ring (group W mean expression), middle ring (group C mean expression), and outer ring (log_2_FC values). Circle sizes correspond to |log_2_FC| magnitudes, with orange/blue hues denoting upregulated/downregulated genes, respectively.

PCA revealed distinct clustering of group W and group C based on transcriptional profiles (Figure 2A), while Venn diagram analysis demonstrated substantial overlap in shared genes alongside group-specific genes in the two groups of samples (Figure 2B). Differential expression analysis identified 154 significantly DEGs, comprising 54 downregulated and 100 upregulated transcripts in group W compare to wild-type group C (Figure 2C). Detailed information of these DEGs is available in Supplementary Table S3. Notably, the top 20 most significantly altered genes (ranked by *p*-value) were primarily enriched in tyrosine metabolism, solute carrier (SLC) gene families, and calcium signaling pathways (Figure 2D).

**Figure 3:**
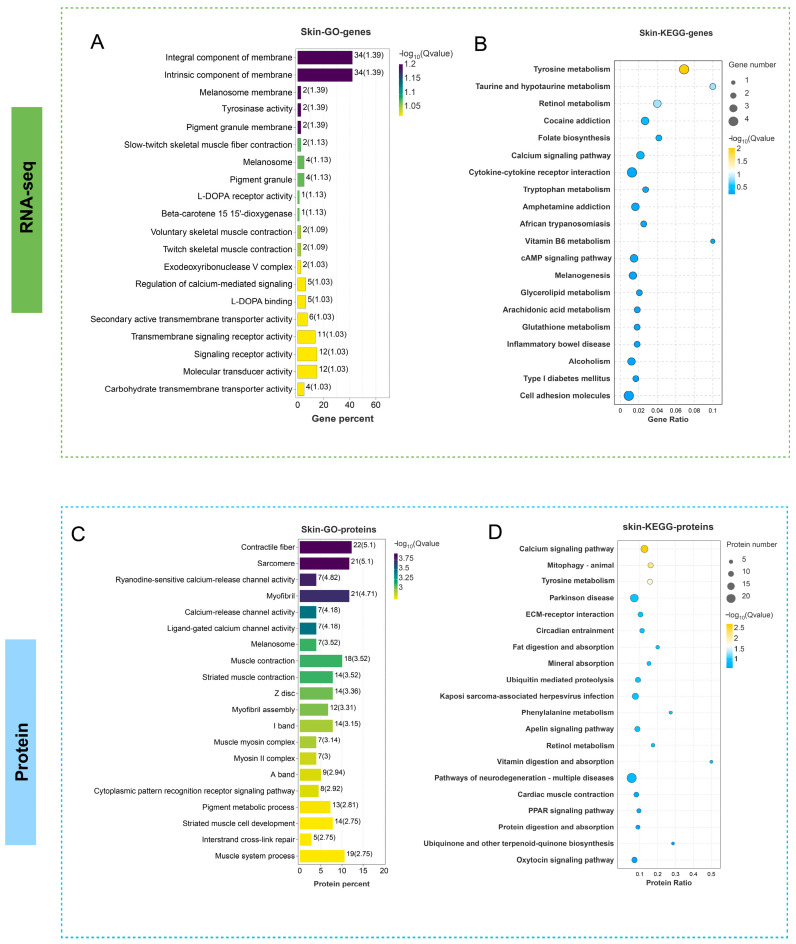
GO enrichment and KEGG enrichment analysis of DEGs in the transcriptome and proteome. (**A**,**B**) GO enrichment and KEGG enrichment analysis of DEGs between two groups in transcriptome, respectively. (**C**,**D**) GO enrichment and KEGG enrichment analysis of DEPs between two groups in proteome, respectively. The vertical axis represents the name of the pathway, and the horizontal axis Rich factor represents the ratio of sample number/background number. The size and color of the dots represent the number of genes and the *p* adjust of each pathway, respectively.

Transcriptomic analysis revealed significant enrichment of DEGs between group W and group C in the following GO categories: melanosome membrane, tyrosinase activity, melanosome, and molecular transducer activity, collectively implicating key biological processes in pigment metabolism, melanosome biogenesis, and ion transport (Figure 3A). KEGG pathway enrichment analysis further demonstrated pronounced DEG convergence in tyrosine metabolism and calcium signaling pathways (Figure 3B).

Proteomic profiling identified DEPs significantly enriched in cytoskeleton-associated GO terms including sarcomere, calcium-release channel activity, ligand-gated calcium channel activity, and myofibril assembly (Figure 3C), while KEGG analysis demonstrated pronounced DEP enrichment in ubiquitin-mediated proteolysis and calcium signaling pathways (Figure 3D).

These integrated omics findings revealed substantial dysregulation across three core pathways in albino *P. pingi* compared to wild-type: melanogenesis, ubiquitin-mediated proteolysis, and calcium signaling pathways, prompting subsequent mechanistic investigations into these regulatory networks.

### 3.3. Analysis of Pathways Related to Melanogenesis

**Figure 4:**
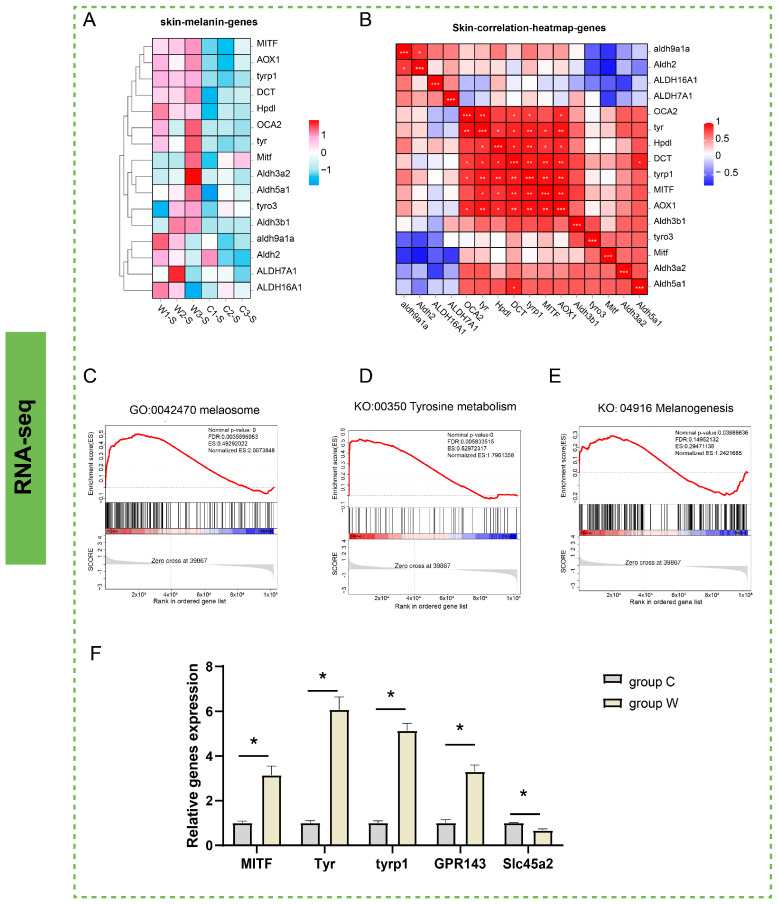
Transcriptomic insights into melanogenesis regulation. (**A**) Z-score normalized heatmap of melanogenesis-associated gene expression across two groups. (**B**) Co-expression network analysis of key melanogenic genes. * denote statistically significant correlations (*p* < 0.05), with more * indicating higher significance levels: *, 0.01 < *p* < 0.05; **, 0.001 < *p* < 0.01; ***, *p* < 0.001. (**C**–**E**) GSEA analysis of the melanosome (GO: 0042470), tyrosine metabolism (KO: 00350), and melanogenesis (KO:04916) in transcriptome, respectively. (**F**) Validation of core regulatory gene expression levels by qRT-PCR (* *p* < 0.05; *n* = 3).

Transcriptomic profiling revealed significant upregulation of most melanogenesis-associated genes in group W compared to group C (Figure 4A). Subsequent correlation analysis demonstrated robust associated relationships among key regulatory genes including *OCA2*, *TYR*, *HPDL*, *DCT*, *TYRP1*, *MITF* and *AOX1* (Figure 4B). GSEA confirmed pronounced activation of melanosome (GO:0042470), tyrosine metabolism (ko00350), and melanogenesis pathway (ko04916) in group W (Figure 4C–E). Quantitative validation via qRT-PCR showed significantly elevated expression of *MITF*, *TYR*, *TYRP1*, and *GPR143* alongside downregulation of the solute carrier gene *SLC45A2* in group W (Figure 4F), consistent with transcriptomic data patterns.

**Figure 5:**
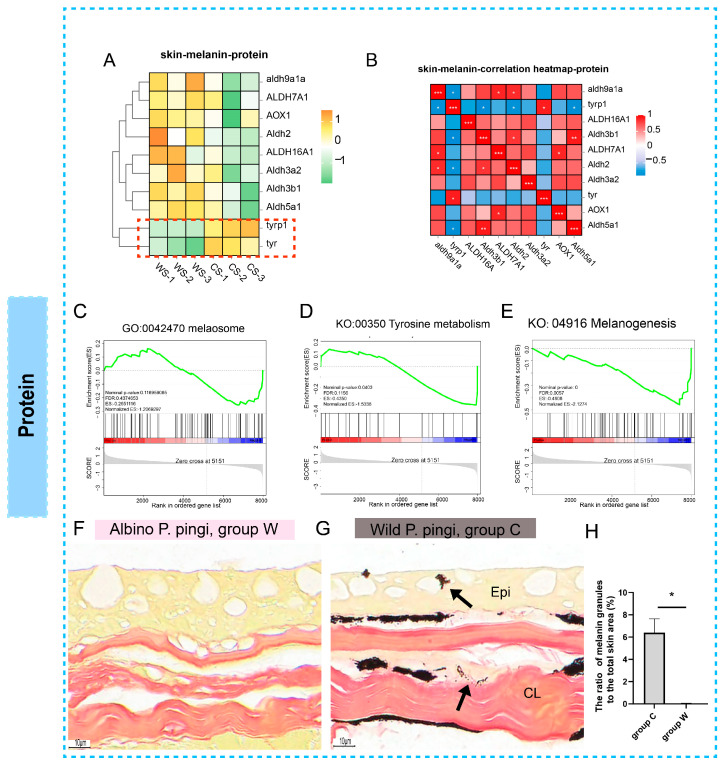
Proteomic profiling of melanogenesis regulation. (**A**) Z-score normalized heatmap of melanogenesis-associated protein expression across groups. (**B**) Co-expression network analysis of key melanogenic protein. * denote statistically significant correlations (*p* < 0.05), with more * indicating higher significance levels: *, 0.01 < *p* < 0.05; **, 0.001 < *p* < 0.01; ***, *p* < 0.001. (**C**–**E**) GSEA at protein level for melanosome (GO:0042470), tyrosine metabolism (KO: 00350), and melanogenesis pathway (ko04916). (**F**,**G**) Fontana–Masson histochemical staining: (**F**) Near-complete absence of melanin granules in group W skin; (**G**) abundant melanin deposition (arrows) distributed across epidermal, dermal, and subcutaneous layers in group C; (**H**) the percentage of melanin granules in the area of the skin (200×, %). Epi, epidermal layer; CL, compact layer.

Proteomic analysis revealed that expression trends of most melanogenesis-associated proteins in group W aligned with transcriptomic profiles. Notably, however, protein levels of the rate-limiting enzymes TYR and TYRP1 were significantly downregulated (Figure 5A). Further analysis demonstrated significantly negative correlations between TYR/TYRP1 and other melanogenic proteins (Figure 5B). GSEA revealed substantial suppression at the protein level for melanosome (GO:0042470), tyrosine metabolism (ko00350), and melanogenesis pathway (ko04916) in the group W (Figure 5C–E).

Histochemical validation via Fontana–Masson staining showed abundant melanin granules distributed from the subcutaneous layer to the epidermis with a diffusion gradient toward the stratum corneum in group C skin (Figure 5G), whereas group W exhibited a near-total absence of melanin deposition (Figure 5F). Quantitative analysis of melanin granules in the skin tissue further confirmed the histological results (Figure 5H). This transcript-protein expression decoupling of TYR/TYRP1 suggests potential dysregulation at translational or post-translational levels.

### 3.4. Analysis of Ubiquitin-Mediated Proteolysis

**Figure 6:**
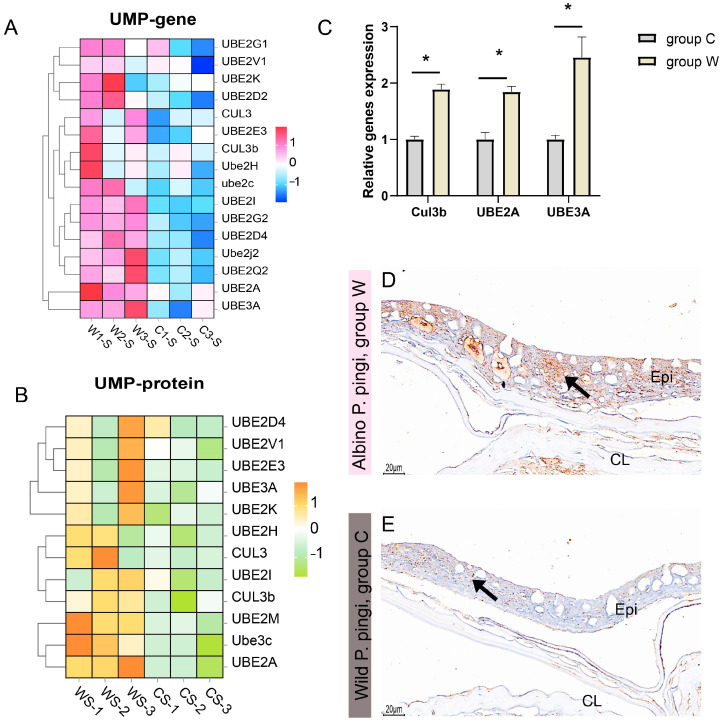
Ubiquitin-mediated proteolysis (UMP) dysregulation. (**A**) Z-score normalized heatmap of UMP-associated gene expression profiles. (**B**) Z-score normalized heatmap of UMP-related protein expression profiles. (**C**) Expression levels of key UMP regulators (*Cul3b*, *UBE2A*, *UBE3A*) validated by qRT-PCR (* *p* < 0.05; *n* = 3). (**D**,**E**) Immunohistochemical (UBE2J1) staining results of skin. The positive results appear brownish-yellow: (**D**) the positive signals (arrows) were strong in group W skin; (**E**) the positive signals (arrows) were weak in group C. Epi, epidermal layer; CL, compact layer.

Integrated transcriptomic and proteomic analyses consistently demonstrated that the key regulatory factors involved in UMP (such as *UBE2A*, *UBE2V1*, *UBE3A*) were significantly upregulated at both mRNA and protein levels in group W compared to group C (Figure 6A,B). Independent qRT-PCR validation further corroborated the elevated expression of core ubiquitin ligase components *Cul3b*, *UBE2A*, and *UBE3A* in group W (Figure 6C). Immunohistochemical staining (UBE2J1) showed that the positive signal in group W was strong and evenly distributed in the epidermis and compact layer (Figure 6D). However, the positive signals in group C were relatively weak and their distribution was more scattered and sparse (Figure 6E).

### 3.5. Analysis of Calcium Signaling Pathways

**Figure 7:**
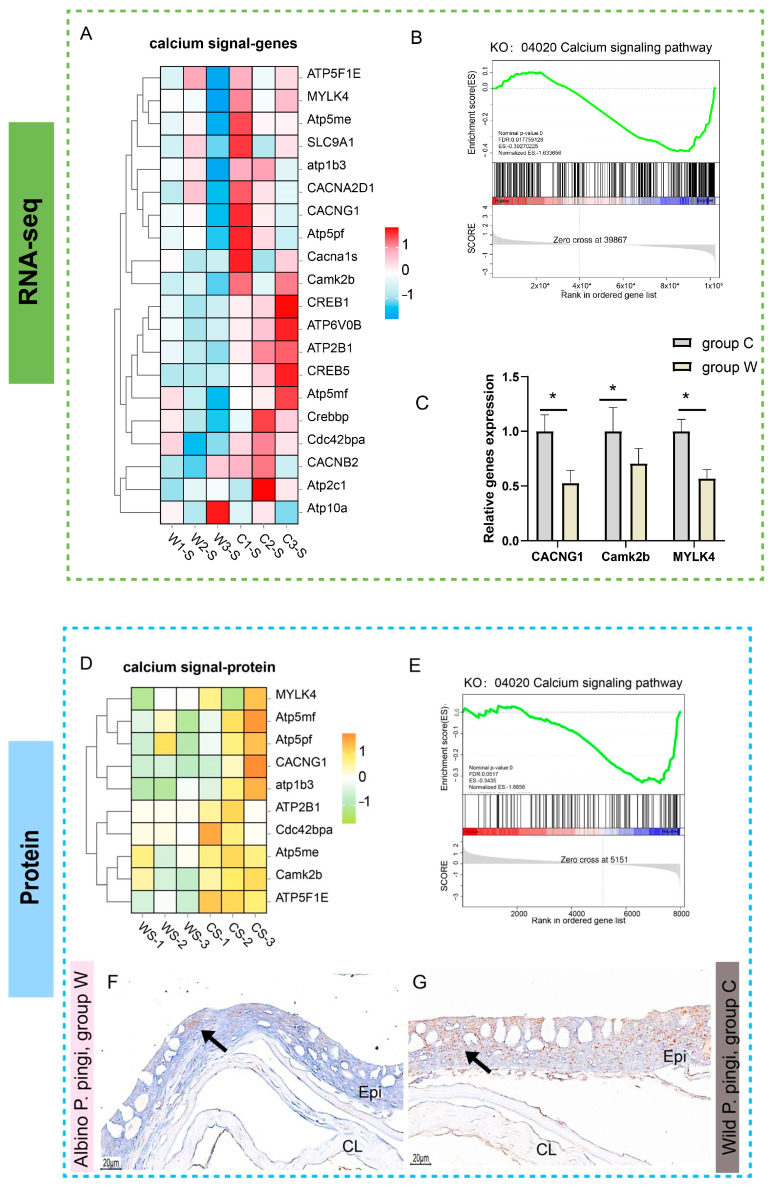
Analysis results related to calcium signaling pathways. (**A**) Z-score normalized heatmap of calcium signaling-associated gene expression profiles. (**B**) GSEA of the calcium signaling pathway (ko04020) in transcriptome. (**C**) Expression levels of key calcium signaling regulators (*CACNG1*, *Camk2b*, *MYLK4*) validated by qRT-PCR (* *p* < 0.05; *n* = 3). (**D**) Z-score normalized heatmap of calcium signaling-related protein expression profiles. (**E**) GSEA of the calcium signaling pathway (ko04020) in proteome. (**F**,**G**) Immunohistochemical (CACNG1) staining results of skin, the positive results appear brownish-yellow: (**F**) the positive signals (arrows) were weak and sparse in group W skin; (**G**) the skin of group C showed strong and dense positive signals. Epi, epidermal layer; CL, compact layer.

Transcriptome analysis and proteome analysis showed a consistent trend. The core components of the calcium signaling pathway (such as MYLK4, TNNC1, and CACNG1) were significantly downregulated at both mRNA and protein levels in group W compared to group C (Figure 7A,D). GSEA further confirmed significant suppression of the calcium signaling pathway (ko04020) in group W at both omics levels (Figure 7B,E), and qRT-PCR validation demonstrated markedly reduced expression of the key regulatory genes *CACNG1*, *Camk2b*, and *MYLK4* in group W (Figure 7C). Immunohistochemical staining (CACNG1) showed that the positive signal in group W was weak and mainly sparsely distributed in the epidermis layer (Figure 7F), while the positive signal in group C was strong and densely distributed in both the epidermis and the compact layer (Figure 7G).

### 3.6. Potential Regulatory Molecular Networks

**Figure 8:**
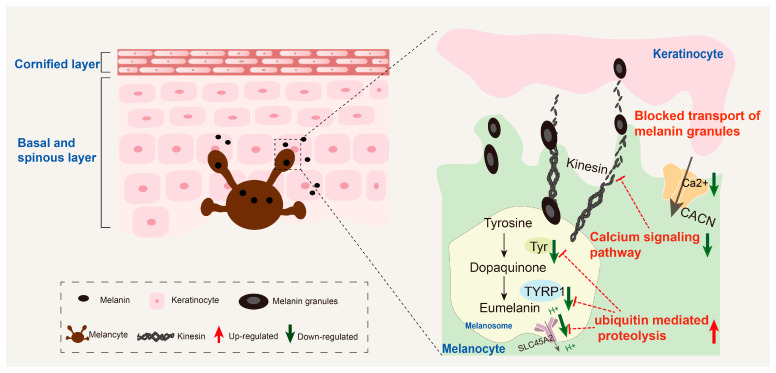
Potential mechanisms of impaired skin melanin synthesis in albino *P. pingi*.

Integrated transcriptomic, proteomic, and molecular biological analyses demonstrate that impaired skin melanin synthesis in albino *P. pingi* is primarily attributed to the synergistic imbalance of two core mechanisms: ubiquitination-mediated degradation disorders and calcium signaling pathway abnormalities (Figure 8). At the ubiquitination level, excessive activation of the UMP pathway significantly downregulates SLC45A2 expression, reducing intracellular H^+^ efflux. The acidic microenvironment disrupts the conformation stability of key enzymes such as TYR and TYRP1, thereby directly impeding the biochemical process of eumelanin synthesis. Concomitantly, misrecognition and degradation of TYR/TYRP1 by the UMP further exacerbate the synthetic impairment. Regarding the calcium signaling pathway, abnormalities occur in CACN-mediated Ca^2+^ transmembrane transport and its downstream cascade signaling. This not only interferes with the transcriptional regulation of melanogenesis-associated genes, but by impairing cytoskeletal dynamics, it also compromises the kinesin–microtubule system-mediated transport of melanin granules to keratinocytes, thereby causing defective pigment deposition.

## 4. Discussion

### 4.1. The Uncoupling Phenomenon of TYR/TYRP1 at the Transcription-Translation Level

TYR serves as the pivotal enzyme in melanogenesis, catalyzing the rate-limiting step of melanin synthesis [24]. Its cofactor, TYRP1, plays a central role in eumelanin production by stabilizing TYR catalytic activity and ensuring melanosomal structural integrity [25]. Consequently, both tyrosinase and TYRP-1 are critically important in regulating melanin biosynthesis.

This study employed integrated multi-omics and molecular biology approaches to systematically dissect the pigment synthesis mechanism in albino *P. pingi.* It was found that compared with the wild-type *P. pingi*, albino individuals exhibited significantly upregulated transcriptional levels of key melanin synthesis-related genes (*OCA2*, *TYR*, *HPDL*, *DCT*, *TYRP1*, *MITF*) in the skin. However, the protein expression of the critical rate-limiting enzymes TYR and TYRP1 was markedly downregulated. This transcription–translation decoupling phenomenon suggests the presence of aberrant post-translational regulatory mechanisms. Proteomic profiling further demonstrated significant enrichment of DEPs between wild-type and albino *P. pingi* within the UMP pathway, demonstrating that the loss of enzyme activity might be caused by abnormal degradation of TYR/TYRP1 via the ubiquitin–proteasome system (UPS).

The pH of melanosomes directly influences TYR activity [26,27]. The catalytic activity of TYR is extremely sensitive to an acidic environment, exhibiting only 20% activity at pH 5.8 compared to pH 6.8 [28]. Given the distinct pH response characteristics of the eumelanin and pheomelanin synthesis pathways, melanosomal pH serves as a core regulatory factor for mixed melanogenesis. Melanosomal pH significantly differs among individuals with varying skin colors; melanosomes are acidic in melanocytes derived from fair-skinned individuals, whereas they approach neutrality in dark-skinned individuals [29]. SLC45A2, a critical regulator of melanosomal pH homeostasis [30], exhibits functional impairment that leads to excessive acidification of early-stage melanosomes, thereby inhibiting TYR activity and limiting melanin synthesis [31].

Consequently, the downregulation of *SLC45A2* observed in this study may have induced intracellular microenvironment acidification, triggering spatial conformational denaturation of TYR. This ultimately created a metabolic bottleneck in melanin synthesis, exacerbating the transcription–translation decoupling effect observed for both TYR and TYRP1, collectively contributing to the formation of the albino phenotype.

### 4.2. Excessive Activation of the UPS

Ubiquitin and ubiquitin-associated proteins regulate protein function through post-translational modification mechanisms. Their dynamic modification processes participate in critical physiological processes, including cell growth, programmed cell death, and DNA damage repair [32]. Research indicates that the stability of TYR depends on its processing and maturation within the endoplasmic reticulum (ER) and Golgi, as well as its degradation via the UPS and/or the lysosomal system [8]. Recent studies have further established that UPS-mediated degradation of TYR constitutes a key regulatory point in modulating skin pigmentation [33]. For instance, Daren et al. [34] demonstrated that dysregulation of the UBE3A interferes with the transcriptional regulatory network of the melanocortin-1 receptor (MC1R), ultimately leading to the hypopigmentation phenotype observed in patients with Angelman syndrome (AS).

In this study, key molecules involved in UMP (including *Cul3b*, *UBE2A*, and *UBE3A*) were significantly upregulated in the albino group, while the expression levels of critical rate-limiting enzymes for melanin synthesis (*TYR*, *TYRP1*) and the *SLC45A2* were markedly downregulated. This pattern suggests aberrant activation of the UPS, likely targeting these key proteins for ubiquitination and degradation via the endoplasmic reticulum-associated degradation (ERAD) pathway, thereby exerting inhibitory regulation on melanin synthesis through reduction of active enzyme content.

### 4.3. Inhibition of the Calcium Signaling Pathway

The transport and accumulation of melanin granules within melanocytes constitute a complex process dependent on the cytoskeletal network formed by microtubules and actin [35,36]. Ca^2+^, acting as second messengers, can activate calmodulin (CaM) and drive actin polymerization and remodeling by regulating the activity of Rho family GTPases (such as Rac1 and Cdc42) [37,38]. Concurrently, alterations in calcium homeostasis regulate microtubule stability and molecular motor activity by influencing the phosphorylation status of the microtubule regulatory factor stathmin. For instance, diminished calcium signaling promotes increased microtubule depolymerization [39,40].

In this study, the expression of cytoskeleton-associated molecules and the calcium signaling pathway (KEGG: 04020) were significantly downregulated at both transcriptional and protein levels in the skin of albino *P. pingi*, indicating substantial suppression of the calcium signaling transduction in this albino model. Under such a low-calcium environment, the transfer of melanosomes to keratinocytes, which was facilitated by the cytoskeleton, was significantly impeded, resulting in melanosome retention within melanocytes. Furthermore, calcium signaling can directly phosphorylate TYR via CaM-dependent kinases such as CaMKII, thereby enhancing its catalytic activity [41]. Consequently, dysregulation of the calcium signaling pathway not only disrupts melanosome transport but also exerts an inhibitory effect on the melanin synthesis process by attenuating TYR activity.

### 4.4. Limitations of This Study and Future Research Directions

Melanin protects organisms from environmental damage, such as resisting ultraviolet damage, antioxidation, and eliminating reactive oxygen species [42]. This protective mechanism may be deeply involved in the regulatory process of growth and development in vertebrates by maintaining the stability of the cellular microenvironment and ensuring signal transduction. This study revealed that albino *P. pingi* not only exhibited the characteristic phenotype of skin pigment deficiency but also displayed significantly lower growth metrics compared to wild-type individuals, including body length and body weight. This growth retardation may be associated with secondary effects of intracellular acidic microenvironment imbalance and impaired calcium signaling transduction, thereby impairing systemic energy metabolism processes.

From a mechanistic perspective, this study, while being the first to propose a synergistic dual-pathway model of “ubiquitination-mediated aberrant protein degradation” and “calcium signaling suppression-induced transport blockade” in fish, provides a novel framework for understanding skin albinism mechanisms in fish.

However, it is important to acknowledge a key limitation of the present work: the relatively small biological replicate size (*n* = 3 per group for sequencing and validation experiments), which was constrained by the endangered status of *P. pingi* and the low natural incidence of the albino phenotype. Although our multi-omics approach provided robust molecular insights, we acknowledge that a larger sample size would have provided greater statistical power to detect more subtle regulatory changes and reduce the potential impact of individual biological variability.

Therefore, future research will be pursued through multiple avenues to further validate and expand upon the current findings: first, collaborating with multiple artificial breeding facilities to collect albino individuals across developmental stages, thereby assessing the generalizability of our conclusions; second, performing single-cell RNA sequencing (scRNA-seq) on the existing samples to dissect cell-type-specific regulatory networks within the skin; and third, utilizing CRISPR/Cas9 to establish albino models in more common and genetically tractable fish species (such as zebrafish), thereby conducting mechanistic dissection of the identified candidate pathways. These integrated approaches are essential to solidify the proposed mechanistic framework and ultimately translate these fundamental discoveries into applications for the molecular breeding of endangered species and the development of therapeutic strategies for pigmentary disorders.

## 5. Conclusions

This study provides evidence supporting the hypothesis that the skin albinism phenomenon in endangered *P. pingi* is caused by the coordinated dysregulation of two core pathways: excessive activation of ubiquitin-mediated protein degradation and inhibition of calcium signal transduction. These dual defects, namely enzymatic depletion and transport blockade, collectively constitute a metabolic bottleneck in the melanin production process, elucidating an unreported mechanism for pigment loss in teleost fishes. This work provides a foundational framework for molecular breeding of endangered species and therapeutic research into pigmentary disorders.

## Data Availability

The RNA-seq data have been deposited in the Genome Sequence Archive (GSA) in National Genomics Data Center, China National Center for Bioinformation/Beijing Institute of Genomics, under the accession number CRA028943.

## Supplementary Materials

The following supporting information can be downloaded at: https://www.mdpi.com/article/10.3390/cells14211684/s1, Table S1. FPKM expression matrix; Table S2. skin-DEGs; Table S3: Primer sequences in this study.
