# Supplementary material for: Multi-Omics Analysis of the Potential Mechanisms of Skin Albinism in Edangered Percocypris pingi: Abnormal Ubiquitination and Calcium Signal Inhibition
Source: Cells. 2025 Oct 27;14(21):1684. doi: 10.3390/cells14211684 (PMC12607500; doi:10.3390/cells14211684)
Supplement: Supplementary file 1 [file cells-14-01684-s001.zip › Table S3. Primer sequences in this study.pdf]

Table S3. Primer sequences in this study.

Gene name	primer sequence (5′-3′)	Reaction Efficiency (%)	GenBank accession number
MITF	F: CTCGCCGTTGTAGTCTCTCC<br>R: GTTGAGGTCCAGAGTGGTGG	98.2	XM_058778880.1
Tyr	F: TTGGTGAGCAGAAGAGGCAT<br>R: TGCATTTTTGAGTGGCTCGG	96.8	XM_058799374.1
Tyrp1	F: GGTGCGACGTAATGTCATGC<br>R: CTGGGCTGGACCCATGTAAG	99.1	XM_058779617.1
GPR143	F: GACACGCTCCTCCACACAAA<br>R: TGCTGTTTCCAGCGGAGAT	97.2	XM_058786580.1
SLC45a2	F: ACTCTGCTATGCCGTTGAGG<br>R: ACGATCGCCCATGTCCTTTT	96.3	XM_058758579.1
CUL3	F: CTCCTCACCAACAAGAGCGT<br>R: GGCCAGTATCCCGTCGTTAG	97.9	XM_058745233.1
UBE3A	F:<br>AGAGCCGCAGAAGAGTGTTAAA<br>R: GAGCCACAAGCCTCATTCGT	97.2	XM_058779676.1
UBE2A	F: GTGGAGCTCCGTCTGAGAAC<br>R: CACAGTTGGGGGCTTGTTTG	98.3	XM_058786115.1
MYLK4	F: CCAACATCTGGAGAACGGCG<br>R: TCTGCGTCTTTCATCTCGTCTT	98.8	XM_058799423.1
CACNG1	F: TACCGGGGGAAATCAACTGC<br>R: GGCACACAGACCTGCAAAAG	98.1	XM_058780099.1
CAMK2b	F:GGAACTTCTCTGTGGGGAGC<br>R: TGATCTCCTGTTTGCGTGCT	96.9	XM_058775357.1
eEF1a	F:ACACAGACTTGGACTGTGCC<br>R: GTAGCTTCACTGCTCGGGAC	99.3	XM_058762838.1
β-actin	TTCAGAGCGTCTTGCCACTT<br>ACGTTCCTCAATCCCAACCC	99.5	JN254630.1
